# How benchmarking of bioinformatics tools is essential for informed workflow selection: a case study on SARS-CoV-2 subgenomic RNA detection

**DOI:** 10.3389/fbinf.2026.1803237

**Published:** 2026-04-22

**Authors:** Gabriele Leoni, Mauro Petrillo, Man-Hung Eric Tang, Soren Alexandersen

**Affiliations:** 1 European Commission, Joint Research Centre (JRC), Ispra, Italy; 2 Seidor Italy S.r.l., Milan, Italy; 3 Department of Sequencing and Bioinformatics, Statens Serum Institut, Copenhagen, Denmark; 4 Department of Animal and Veterinary Sciences, AU-Viborg, Research Centre Foulum, Aarhus University, Tjele, Denmark; 5 School of Medicine, Deakin University, Geelong, VIC, Australia

**Keywords:** benchmarking, bioinformatics, NGS, SARS-CoV-2, sgRNAs

## Abstract

**Introduction:**

Selecting appropriate bioinformatics tools is critical for accurate and reproducible analysis, particularly in support of genomic surveillance and molecular biomarker monitoring. The importance of these analyses is underscored by the need for effective public health responses to emerging diseases like SARS-CoV-2.

**Methods:**

By using the detection of SARS-CoV-2 subgenomic RNAs (sgRNAs) as a case study, we show the importance of systematic benchmarking in selecting optimal workflows. We generated 25 synthetic Illumina datasets simulating both shotgun and amplicon sequencing strategies, along with a real-world wastewater dataset. Using these datasets, we assessed the influence of key variables including mutation profiles, read lengths, aligner choice, and primer design for targeted sequencing.

**Results:**

Our results revealed substantial performance variability: common tools developed to identify sgRNAs struggled with shotgun data and were sensitive to mutations depending on the chosen aligner, while amplicon sequencing improved detection sensitivity, with aligners and primer design choices still significantly impacting outcomes.

**Discussion:**

Our results highlight the need for benchmarking steps and analyses to inform workflow selection. Without such evaluations, researchers risk drawing inaccurate conclusions from suboptimal workflows. This case study underscores the value of context-aware tool selection and encourages standardised benchmarking practices to ensure reproducibility and reliability in bioinformatics analysis, particularly in evidence-based decision-making environments such as public health and policymaking.

## 1 Introduction

The rapid growth of bioinformatics tools and pipelines has revolutionised the field of biological data analysis, offering researchers unprecedented capabilities to explore omics landscapes (Di Tommaso et al., 2017). However, this proliferation has introduced a critical challenge. Selecting the most appropriate computational methods for a given biological question and increasing the reproducibility of computed results are of paramount importance (Cohen-Boulakia et al., 2017). As digital objects to be shared, discovered, and reused, computational workflows, including bioinformatics ones, benefit from the FAIR principles, namely, Findable, Accessible, Interoperable, and Reusable (Wilkinson et al., 2025). Benchmarking, i.e., the systematic evaluation of tool performance under controlled conditions, is a crucial strategy to guide informed workflow selection, ensure reproducibility, and promote methodological transparency (Weber et al., 2019). Without rigorous benchmarking, there is risk that researchers may draw inaccurate conclusions from suboptimal or misused tools in complex biological contexts (Mangul et al., 2019). The COVID-19 pandemic underscored the critical importance of benchmarking bioinformatics tools, particularly in the context of genomic surveillance for public health. This framework provides a rich source of information for tracking pathogen transmission and evolution on both national and international levels (Lo and Jamrozy, 2020) and the incentive of sharing massively high-quality genomic sequence data with fair and equitable sharing of benefits as recommended by the Nagoya Protocol (Scholz et al., 2022). Genomic surveillance has become the preferred method for monitoring and studying this fast-spreading virus and its constantly emerging lineages (Charre et al., 2020). The accurate, comparable, and reproducible detection of viral variants and lineage assignment across diverse sequencing platforms and protocols depends on the rigorous evaluation of computational workflows (Connor et al., 2024).

In evidence-based decision-making environments such as public health and policymaking, accurate and reproducible bioinformatics workflows are essential for genomic surveillance of pathogens with epidemic or pandemic potential. The One Health approach, which integrates human, animal, and environmental health to optimise outcomes and prevent zoonotic spillovers (https://www.who.int/news-room/q-and-a/detail/one-health), underpins global efforts in this domain. The World Health Organization’s Global Genomic Surveillance Strategy for Pathogens with Pandemic and Epidemic Potential (2022–2032) provides a 10-year framework to strengthen and integrate genomic surveillance into public health systems worldwide, explicitly calling for enhanced capacities including bioinformatics tools and data analysis (WHO global genomic surveillance strategy for pathogens with pandemic and epidemic potential 2022-2032). In this context, the One Health High-Level Expert Panel (OHHLEP) has emphasised the need for structured surveillance systems that incorporate robust data analysis and interpretation methods (Hayman et al., 2023). Systematic benchmarking of bioinformatics tools, as undertaken in this study, contribute to these goals by enabling reliable, context-aware detection of molecular markers like sgRNAs, thereby improving the evidence base for surveillance and response.

### 1.1 Subgenomic RNAs (sgRNAs) as a case study: biological relevance and detection challenges

SgRNAs are produced during viral replication through a discontinuous transcription mechanism involving template switching at transcription regulatory sequences (TRSs) (Sawicki et al., 1995; Sola et al., 2015). The SARS-CoV-2 genome is a positive-sense single-stranded RNA (gRNA). During negative-strand synthesis, negative-sense intermediates known as antigenome RNAs (agRNAs) serve as templates for positive-sense gRNAs and sgRNAs. Formation of sgRNAs is regulated by cis-acting TRSs, short sequences (6–12 nucleotides) containing a conserved core motif (ACGAAC in SARS-CoV-2) flanked by variable regions. TRSs are located in the 5′untranslated region (UTR) as leader TRSs (TRS-Ls) and near the start codons of open reading frames (ORFs) encoding structural and accessory proteins in the distal third of the genome (body TRSs or TRS-Bs). During negative-strand synthesis, the replicase–transcriptase complex pauses at a TRS-B complementary to TRS-L, triggering template switching by the RNA-dependent RNA polymerase and producing antisense sgRNAs that fuse the 5′leader with downstream body sequences. These are then transcribed into a nested set of positive-sense sgRNAs sharing the same 5′leader (Alonso et al., 2002; Hiscox et al., 1995; La Monica et al., 1992; van Marle et al., 1995; Thiel et al., 2003). Beyond canonical sgRNAs encoding functional proteins, non-canonical sgRNAs arise from atypical events such as truncated fusions, frameshifted ORFs, and body-to-body junctions (Long, 2021; Masters, 2006; Ozdarendeli et al., 2001). Despite their association with active viral genome expression, the utility of sgRNAs as diagnostic markers of ongoing infection remains controversial. Early studies using PCR-based assays, such as Wölfel et al. (2020), which targeted sgRNAs encoding for the envelope (E) protein, reported a positive correlation between sgRNA detection and viral replication in clinical samples. In contrast, Alexandersen et al. (2020) applied next-generation sequencing (NGS) combined with a dedicated bioinformatics pipeline to clinical specimens and observed that sgRNAs can persist after the period of active replication. This work represented an early attempt to detect and quantify SARS-CoV-2 sgRNAs using bioinformatic approaches on NGS data. These conflicting findings highlight ongoing uncertainty about the biological and clinical interpretation of sgRNA detection and emphasise the need for robust, standardised methodologies to clarify their role.

Diverse sequencing approaches were used during the pandemic, including IonTorrent and Illumina amplicon sequencing (Alexandersen et al., 2020; Parker et al., 2021; Wong et al., 2021), Nanopore direct sequencing (Davidson et al., 2020; Taiaroa et al., 2020), poly(A) enrichment (Dong et al., 2022; Finkel et al., 2021; Thorne et al., 2022), metagenomics (Thorne et al., 2022), and capture-based methods (Parker et al., 2021; Thorne et al., 2022), with amplicon protocols becoming dominant. While this allowed the massive generation of sequencing data suited for genomic surveillance and the reporting of circulating variants, the development of reliable computational detection tools of sgRNAs, particularly canonical junctions, was hindered by several inherent biological and experimental challenges.

First, the SARS-CoV-2 leader sequence is short (∼70–75 nucleotides from the 5′end to the TRS-L core) (Davidson et al., 2020; Kim et al., 2020), making reads mapping this region more vulnerable to mutations, incomplete 5′coverage from RNA degradation, or biases in library preparation that can lead to under-detection of junctions.

Secondly, canonical junction detection requires reads to span across the leader-body fusion. Shorter reads tend to be more sensitive to sequence variations near junctions, limiting robust alignment across lineages, whereas longer reads (≥150–300 bp paired-end) enhance spanning and mutation tolerance in both shotgun and amplicon data (Parker et al., 2021; Dong et al., 2022; Cohen et al., 2023). Lastly, in amplicon-based sequencing, the predominant method for SARS-CoV-2 surveillance, primer placement critically affects sgRNA evidence. Forward primers must cover sufficient leader sequence upstream of TRS-L, and reverse primers must enable downstream coverage of TRS-B regions. Suboptimal placement (e.g., forward primers starting too close to TRS-L) can exclude or misalign leader-containing reads, artificially reducing signals (Parker et al., 2021; Baudeau and Sahlin, 2024). Iterative amplicon generation protocols (i.e., ARTIC versions v.4 vs. v5.3.2) have improved 5′coverage and reduced dropout from primer-site mutations (Baudeau and Sahlin, 2024). Real-world samples often harbour mutations near TRS sites or mixed lineages with varying frequencies, altering transcription efficiency or aligner performance and introducing biases (Thorne et al., 2022; Leary et al., 2021; Tang et al., 2023).

These constraints have driven the development of specialised bioinformatics tools capable of identifying canonical and non-canonical junctions from virus-specific reads. However, detection and quantification remain highly dependent on sequencing strategy, particularly primer positioning relative to the leader and TRS-Bs and the choice of the sequence read length required to span across the canonical junctions. Only a few tools exist for this purpose, including LeTRS (Dong et al., 2022), Periscope (Parker et al., 2021), sgDI-tector (Di Gioacchino et al., 2022), and the sgRNAdetect pipeline used in Tang et al. (2023) (Table 1). As noted by Mori and colleagues (Mori et al., 2023), their scarcity is compounded by limited benchmarking data. One preliminary comparison showed similar quantification across tools (Lavezzari et al., 2023), but it was restricted to a single lineage without critical mutations and did not evaluate performance across sequencing protocols or experimental variations, limitations that preclude robust assessment of tool reliability in diverse contexts.

**TABLE 1:** Comparison of sgRNA detection tools. Summary of features, strengths, limitations, and peculiarities of four bioinformatics tools (sgDI-tector, Periscope, LeTRS, and the sgRNAdetect pipeline) used for SARS-CoV-2 subgenomic RNA (sgRNA) detection. Attributes include input data requirements, alignment methods, TRS dependency, supported sequencing types, and adaptability to other viruses.

Attribute	LeTRS	Periscope	sgDI-tector	sgRNAdetect
References	Dong et al., 2022	Parker et al., 2021	Di Gioacchino et al., 2022	Tang et al., 2023 - (https://github.com/ssi-dk/project-covid19_subgenomic)
Input data (read layout and platform)	FASTQ (single-end or paired-end, platform-dependent) or BAM	FASTQ (raw paired-end; illumina, nanopore ARTIC)	FASTQ (single-end; any platform)	BAM (from paired-end FASTQ; illumina)
Alignment method	HISAT2	BWA	BWA	BWA
TRS dependency	Yes (leader-dependent)	Yes (leader-dependent)	No (leader-independent)	Yes (leader-dependent)
sgRNA detection	Perl pipeline identifies leader–TRS junctions; searches outside intervals for nc-sgRNA	Local alignment to identify leader-containing reads; TRS-based	Detects fragmented reads (insertions, deletions, copy-back, hairpins) using DI-tector; prioritises abundant ORFs	Requires 5′ UTR mapping with mate in gene regions; breakpoint identification
Supported sequencing	Amplicon ARTIC, direct RNA, paired-end (any platform)	Metagenomic, bait-capture, amplicon-based ARTIC (illumina, nanopore)	Metagenomic, single-end, any platform	Amplicon ARTIC (illumina)

These biological and experimental realities underscore the need for systematic benchmarking. The present study addresses this by isolating the effects of key variables, mutation profiles, read lengths, aligner choice, and primer scheme, on sgRNA detection workflows.

## 2 Methods

### 2.1 Tool selection

Four pipelines were selected for their prominence in sgRNA detection studies (Tang et al., 2023; Lavezzari et al., 2023):

- LeTRS (v. 2.2.1).
- Periscope (v. 0.1.2).
- sgDI-tector (April 2025 version).
- sgRNAdetect (https://github.com/ssi-dk/project-covid19_subgenomic).

All tools were used with default parameters unless specified in the text.

### 2.2 Variables and dataset characteristics

The variables on the *in silico* detection and quantification of sgRNAs from the gene N encoding for the nucleocapsid protein of SARS-CoV-2 included:

- Sequencing strategy i.e., shotgun and amplicon.
- Library strategy i.e., ARTIC v.4 and ARTIC v.5.3.2, https://github.com/artic-network/artic-ncov2019/tree/master, limited to the amplicon sequencing strategy.
- Sequencing instrument and errors models i.e., MiSeq and HiSeq.
- Read length i.e., 125 bp (HiSeq) and 300 bp (MiSeq).
- Aligner for mapping of reads on the used reference i.e., BWA (Li and Durbin, 2009) and HISAT2 (Kim et al., 2019).
- Presence of mutations i.e., no mutations, one mutation in the TRS-B sequence, one mutation in the N gene, two mutations in the N gene, and three mutations in the N gene.

**FIGURE 1:**
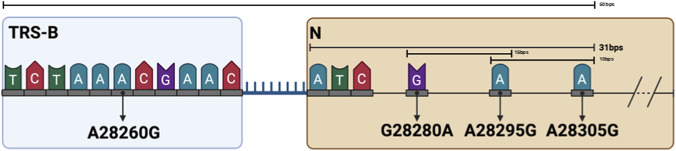
Schematic of the SARS-CoV-2 N gene region highlighting the transcription regulatory sequence-body (TRS-B) and introduced mutations. The TRS-B sequence is located immediately upstream of the N gene start codon (position 28,274). The A28260G mutation is perfectly in the middle of the TRS-B region. The G28280A, A28295G and A28305G mutations are located within the early coding sequence of the N gene (6 bp, 21 bp and 31 bp downstream of the start codon, respectively). Overall, these mutations span approximately 45 nucleotides, allowing even 125 bp sequencing reads to simultaneously cover the TRS-B region and the N gene mutations.

The following mutations were considered: (i) A28260G within the TRS-B region of the N gene, (ii) G28280A, (iii) G28280A and A28295G and (iv) G28280A, A28295G and A28305G which are within the 5′ region of the gene N. These mutations, although not frequently observed in real samples, were specifically selected for their particular positions. A28260G is precisely located in the middle of the TRS-B while G28280A, A28295G and A28305G are placed within the initial 75 bps of the N gene (the selected read length for the sgRNAs supporting reads in the shotgun analyses), with sufficient distance from each other’s (Figure 1). To investigate the impact of these variables on the tools’ performances, we generated 25 synthetic datasets using InSilicoSeq v. 2.0.1 (Gourlé et al., 2019) (Table 2), available at https://doi.org/10.2905/JRC.1RYH4D0.

**TABLE 2:** Synthetic datasets for benchmarking SARS-CoV-2 sgRNA N detection. Description of 25 in silico-generated datasets used to evaluate sgRNA N detection. Datasets vary by sequencing strategy (shotgun, n = 5; amplicon, n = 20), library type (ARTIC v.4 vs. v.5.3.2 for amplicon), error model (MiSeq vs. HiSeq), read length (125 bp vs. 300 bp), and mutation profiles (wild-type, TRS-B mutation, and 1–3 N gene mutations). Each dataset includes 500,200 reads, with 100 paired-end reads designed to support or not support sgRNA N events.

Number of datasets	Sequencing strategy	Sequencing library	InSilicoSeq error model	Read length (bps)	Mutation profiles
5	Shotgun	NA	Basic	125, sgRNA supporting reads = 75	- (WT)
					A28260G (TRS-B)
					G28280A (N)
					G28280A, A28295G (N)
					G28280A, A28295G, A28305G (N)
5	Amplicon	ARTIC v.4	MiSeq	125	- (WT)
					A28260G (TRS-B)
					G28280A (N)
					G28280A, A28295G (N)
					G28280A, A28295G, A28305G (N)
5	Amplicon	ARTIC v.4	HiSeq	300	- (WT)
					A28260G (TRS-B)
					G28280A (N)
					G28280A, A28295G (N)
					G28280A, A28295G, A28305G (N)
5	Amplicon	ARTIC v.5.3.2	MiSeq	125	- (WT)
					A28260G (TRS-B)
					G28280A (N)
					G28280A, A28295G (N)
					G28280A, A28295G, A28305G (N)
5	Amplicon	ARTIC v.5.3.2	HiSeq	300	- (WT)
					A28260G (TRS-B)
					G28280A (N)
					G28280A, A28295G (N)
					G28280A, A28295G, A28305G (N)

### 2.3 Shotgun synthetic datasets

Five synthetic shotgun datasets were constructed. The wild-type shotgun dataset comprised 500,200 reads. 500,000 of them (250,000 paired-end reads) did not support sgRNAs and were generated using InSilicoSeq with the reference genome wuhCor1 (Wu et al., 2020) and the following options: [command: generate, options: --n_reads 500000 –compress–mode basic–genomes WuhCor1.fa]. Additionally, 100 paired-end reads (200 in total) were manually designed and grouped into three sets with distinct properties: (a) 10 paired-end reads spanning positions 1 to 75, with mates spanning positions 28,350 to 28,424; (b) 74 paired-end split reads starting at variable positions within the 5′ UTR (using a sliding window) and ending within the N gene, with mates spanning positions 28,350 to 28,424; (c) 26 paired-end reads spanning positions 28,255 to 28,329, with mates spanning positions 28,350 to 28,424. Of these, only group c) does not support sgRNA N events. Four additional SARS-CoV-2 genomes were generated to incorporate the following mutations previously described. Using these mutated genomes, four additional synthetic shotgun datasets were created by applying the same InSilicoSeq read generation procedure as described for the wild-type dataset.

### 2.4 Amplicon synthetic datasets

Twenty synthetic amplicon datasets were generated, using InSilicoSeq (options: [command: generate, option: --genomes amplicons.fasta--readcount_file amplicon.counts.txt -sequence_type amplicon–model miseq/hiseq]). For each of the five SARS-CoV-2 genomes produced for the shotgun datasets, we created ARTIC v.4 and ARTIC v.5.3.2 versions by providing the respective primer schemes (obtained from: https://github.com/artic-network/primer-schemes/tree/master/nCoV-2019). We generated 500 read pairs for each amplicon providing a read count file to InSilicoSeq. For each library version, datasets were generated for both Illumina HiSeq and MiSeq instruments models, resulting in 20 distinct datasets. To incorporate evidence of sgRNA N, we modified the primer schemes by adding 100 sgRNA-supporting artifact amplicons. Details of the added amplicons are reported in Table 3.

**TABLE 3:** sgRNA-supporting artifact amplicon primer scheme. Details of ARTIC prime scheme modifications to simulate sgRNA N amplification in amplicon datasets. The scheme includes forward and reverse primers for ARTIC v.4 (positions 25–64 and 28,254–28,598) and v.5.3.2 (positions 47–64 and 28,254–28,873), linked to read count files for InSilicoSeq.

ARTIC version	Primer type	Start position (bp)	End position (bp)
v.4	Forward	25	64
v.4	Reverse	28,254	28,598
v.5.3.2	Forward	47	64
v.5.3.2	Reverse	28,254	28,873

### 2.5 Wastewater dataset

The wastewater dataset consists of 1,013 samples (29 from a pilot study) from the Danish SARS-CoV-2 wastewater surveillance program (Krogsgaard et al., 2024), collected from August 2023 until December 2023 (Study accession: PRJEB65603). Paired-end sequencing was performed using Illumina NextSeq 550 instrument at the Statens Serum Institut. The first samples with ID ERS16306335-ERS16306450 that have been sequenced using the ARTIC v.3 primer scheme with in-house modifications were not included in the analysis. Remaining samples were sequenced using the ARTIC v.5.3.2 primer scheme. Samples were processed with PathoSeq-QC (v. 0.1, parameters: --SARS_CoV_2) (Leoni et al., 2025) and mutations were extracted from the GATK results.

### 2.6 Tool selection, adaptation, and performance evaluation

We evaluated the performance of three sgRNA detection tools, LeTRS, Periscope, and sgDI-tector, alongside the sgRNAdetect pipeline, using Illumina paired-end sequencing data generated *in silico* with default parameters. Due to inherent incompatibilities, each tool was adapted to ensure compatibility with the datasets. While LeTRS, sgDI-tector, and the sgRNAdetect natively supported Illumina sequencing data, Periscope code was modified to incorporate a custom XC:Z field into its output BAM file to flag sgRNA-supporting reads. This modification facilitated visual inspection of detection accuracy using the Integrative Genomics Viewer (IGV) (Robinson et al., 2011) (v.2.19.4). For sgDI-tector, paired-end sequencing data compatibility was achieved by concatenating read and mate files into a single input file. LeTRS analyses can accept BAM or Fastq files as input source. While analysis of BAM files requires a single step, processing Fastq files involves two steps: a splice-aware mapping step using HISAT2, followed by sgRNAs detection. However, the second step requires a primer scheme file, which is not applicable to shotgun data. To address this, we performed the first HISAT2 mapping step independently to generate BAM files, which were then used as input for LeTRS BAM-based analysis. The resulting count files from all tools were validated by manually inspecting supporting reads in BAM files using IGV. The HISAT2 mapped reads were additionally used as input for Periscope and for sgRNAdetect to assess the impact of different mappers on their performance.

For shotgun datasets, performance was assessed by considering reads and mates individually, as both discordant and split reads can indicate sgRNA presence, yielding a total of 148 supporting reads, out of 200, for analysis. For amplicon datasets, read pairs were treated as single units, as individual reads and mates cannot span both the 5′ UTR and N gene, and only discordant pairs can support sgRNA events. This approach limited the total number of units to 50.

For wastewater samples, as the four tools employ heterogeneous output formats and normalisation strategies, raw junction-supporting read counts (LeTRS, sgRNAdetect), structural variant read counts (sgDI-tector), and cluster-level abundance scores (Periscope), no common numerical threshold is applicable across tools. A sample was therefore classified as positive for a given sgRNA species if the tool reported at least one detected event for that species, irrespective of the associated numerical value. This qualitative criterion corresponds to the minimum detectable signal under each tool’s own output format and is consistent with the exploratory scope of the wastewater analysis.

### 2.7 Bioinformatics analysis and visualisation

Statistical analyses and visualisations were performed in R (v. 4.5.0) using standard libraries and the ggplot2 package (v. 3.5.2).

## 3 Results

### 3.1 Dataset resource

One major challenge in benchmarking sgRNA detection tools for SARS-CoV-2 is the lack of standardised output formats and quantification metrics, compounded by diverse normalisation strategies (e.g., reads per genomic RNA, per total mapped reads, or peak/cluster-based counts) and differing sensitivity to noncanonical junctions. These variations lead to discordant abundance estimates, for tools such as Periscope, LeTRS, and sgDI-tector, particularly for low-abundance or noncanonical sgRNAs (Baudeau and Sahlin, 2024; Lavezzari et al., 2023). To enable robust benchmarking, we generated 25 synthetic Illumina datasets that simulate diverse shotgun and amplicon scenarios with controlled mutation profiles, aligners, and primer scheme. These datasets, which are publicly available at European Commission and Joint Research Centre (2026), provide a standardised resource for researchers to evaluate and develop sgRNA detection tools, addressing the scarcity of benchmarking data noted by Mori et al. (2023).

### 3.2 Analysis on shotgun sequencing strategy datasets

**FIGURE 2:**
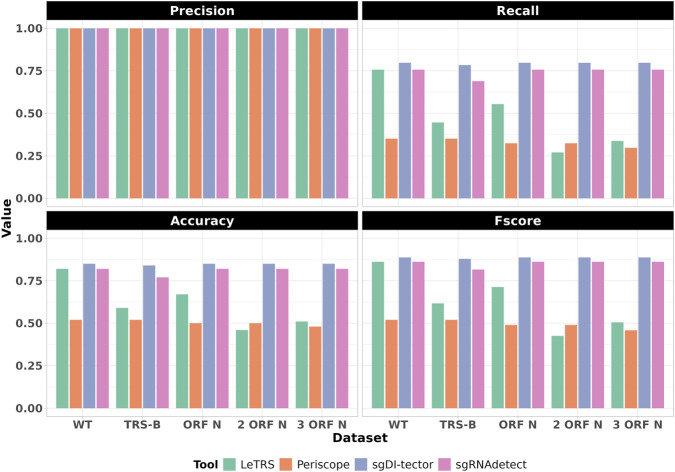
Performance metrics for shotgun sequencing datasets. Bar plots comparing Precision, Recall, Accuracy, and F-score for LeTRS, Periscope, sgDI-tector, and sgRNAdetect (Tang et al., 2023) across five shotgun datasets (wild-type, TRS-B mutation, and 1–3 N gene mutations). Results highlight tool variability and the impact of mutation profiles on sgRNA N detection.

We evaluated the four tools using five synthetic shotgun sequencing datasets, representing five distinct SARS-CoV-2 genomes: a wild-type (WT) genome, a TRS-B mutated genome (A28260G), a genome with a single N gene mutation (G28280A), a genome with two N gene mutations (G28280A and A28295G), and a genome with three N gene mutations (G28280A, A28295G, and A28305G). Each dataset comprises 500,000 paired-end reads of 125 bps in length that do not support sgRNAs, and 100 paired-end reads (200 total reads) of 75 bps in length designed that included both discordant and split reads. As described in the Methods section, these reads were designed to include or exclude sgRNA N events. Tool performance was measured by their ability to detect the 74 True Positive sgRNA-supporting reads pairs, including both discordant and split reads as evidence of sgRNA events (Figure 2).

Performance varied markedly across tools, influenced by mutation profiles and alignment algorithms (Supplementary Table S1), but most notably, no tool was able to detect all sgRNA supporting reads.

Periscope consistently underperformed across all datasets (mean precision: 1, mean recall: 0.33, mean accuracy: 0.50 and mean Fscore: 0.50), with stable but suboptimal results regardless of mutations (standard deviations: 0, 0.023, 0.017, 0.026, respectively).

In contrast, sgDI-tector exhibited the highest performance across all conditions (mean precision: 1, mean recall: 0.80, mean accuracy: 0.85, and mean Fscore: 0.89).

SgRNAdetect performed comparably to sgDI-tector (mean precision: 1, mean recall: 0.74, mean accuracy: 0.81, and mean Fscore: 0.85), maintaining stability across most conditions. However, an exception occurred with the TRS-B mutation (A28260G), where a decrease in performance was noted, suggesting that the pipeline’s dependence on precise mapping to the 5′UTR and N gene regions is sensitive to TRS alterations and therefore to robust aligner tools.

In the wild-type (WT) dataset, LeTRS exhibited performance comparable to sgDI-tector and sgRNAdetect, achieving high precision, recall, accuracy, and F-score (precision: 1, recall: 0.76, accuracy: 0.82 and Fscore: 0.86). Its performance declined markedly in datasets containing mutations, particularly those with two or three mutations in the N gene. In these datasets, LeTRS’s metrics approached those of Periscope (mean precision: 1.00, mean recall: 0.47, mean accuracy: 0.61, and mean F-score: 0.62).

Analysis of BAM files with planted mutations in the N gene locus revealed that HISAT2 mapped fewer reads than BWA when multiple mutations were present (Supplementary Figure S1). However, HISAT2 accurately aligned reads to the 5′untranslated region (UTR) and up to the transcription-regulatory sequence leader (TRS-L) region, consistent with its splice-aware design. In contrast, BWA misaligned reads originating from position 46, flagging them as split reads and erroneously mapping them to the N gene locus (Supplementary Figure S2).

To further investigate this finding, we applied sgRNAdetect to HISAT2-mapped reads. In the WT dataset, this resulted in an almost perfect performance (Precision: 1, Recall: 0.99, Accuracy: 0.99, Fscore: 0.99), as the ability to correctly include discordant reads information were strengthened by the ability of HISAT2 in correctly mapping all 5′UTR reads. However, in datasets with mutated sequences, performance declined significantly (Supplementary Figures S3, S4). A summary of performance variability across all mutation conditions, presented as mean ± SD of Recall, F-score, and Accuracy per tool, is provided in Supplementary Figure S5.

We hypothesised that these challenges may be exacerbated in amplicon sequencing due to constraints imposed by the ARTIC primer design in the 5′ UTR region and how alignment algorithms handle reads partially mapping outside of the first amplicon (see Methods, Table 3). This prompted further investigation into amplicon sequencing datasets to assess the generalisability of our findings.

### 3.3 Analysis on amplicon sequencing strategy datasets

To strengthen the observations made with the shotgun dataset analyses, and to examine the influence of additional variables, amplicon primer scheme (ARTIC v.4 vs. v.5.3.2), read length (125 bp vs. 300 bp), error model (HiSeq vs. MiSeq), we analysed the tools’ performance on 20 additional synthetic amplicon datasets. For these datasets, which are based on the same five SARS-CoV-2 genomes as the shotgun datasets, we treated read pairs as single units as due to the constraint imposed by the amplicon sequencing, only forward reads might be used in supporting sgRNA N events, yielding a maximum of 50 supporting units per dataset.

**FIGURE 3:**
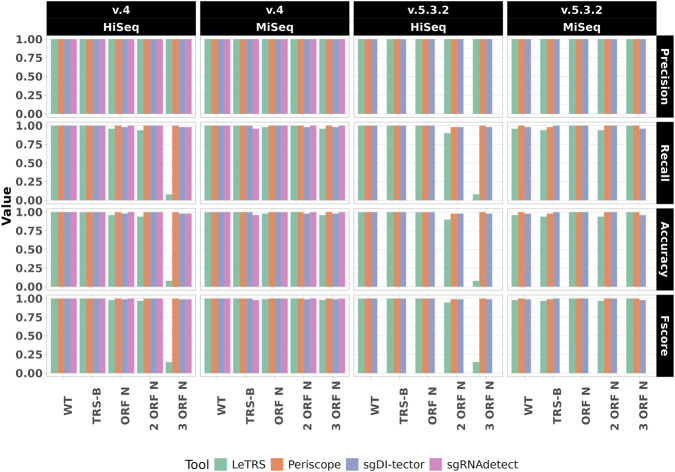
Performance metrics for amplicon sequencing datasets. Bar plots illustrating precision, recall, accuracy, and F-score for LeTRS, Periscope, sgDI-tector, and sgRNAdetect across 20 amplicon datasets, grouped by ARTIC version, read length/error model, and mutation profiles. The figure demonstrates improved performance in amplicon sequencing and the influence of mutations for LeTRS. SgRNAdetect results presented here are based on BWA alignments. For a stratified comparison with HISAT2, see Figure 4, which highlights aligner-specific differences, particularly the negative impact of BWA on ARTIC v.5.3.2 datasets.

Overall, tool performance improved compared to shotgun datasets, reflecting the targeted nature of amplicon sequencing (Figure 3; Supplementary Table S2). sgDI-tector maintained top performances across all amplicon datasets (mean Precision: 1, mean Recall: 0.99, mean Accuracy: 0.99 and mean Fscore: 0.99), demonstrating its versatility across sequencing strategies. Periscope showed enhanced performance relative to shotgun data with BWA (mean Precision: 1, mean Recall: 1, mean Accuracy: 1, mean Fscore: 1), with both ARTIC versions and instrument models. However, when applied to HISAT2-mapped data, Periscope’s performance dropped to 0 due to the absence of split reads, a consequence of HISAT2’s splice-aware mapping behaviour.

**FIGURE 4:**
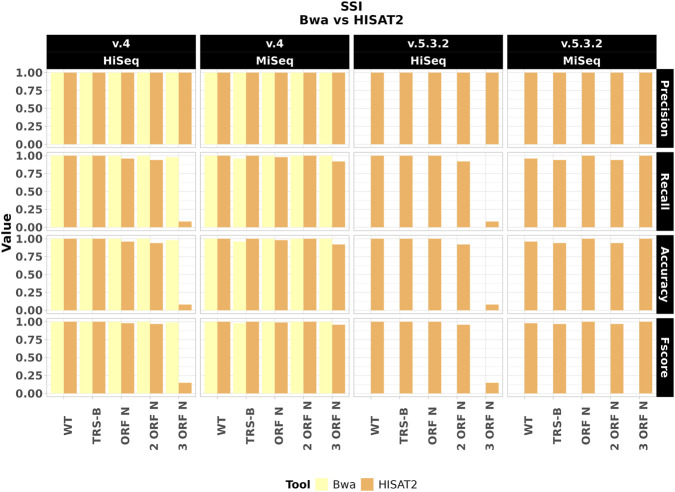
Performances of sgRNAdetect on amplicon data depend on the chosen alignment. Barplot illustrating Precision, Recall, Accuracy and F-score for sgRNAdetect across 20 amplicon datasets, grouped by ARTIC version, read length/error model, and mutation profiles. Coloured bars reflect the different alignment used (BWA or HISAT2), evidencing how BWA aligned data negatively influenced sgRNAdetect performances with ARTIC v.5.3.2 version, while HISAT2 mapped data are not affected.

SgRNAdetect exhibited good performances with HISAT2-mapped reads (mean Precision: 1, mean Recall: 0.89, mean Accuracy: 0.89, mean Fscore: 0.90) but failed to detect sgRNA evidence when using BWA with ARTIC v.5.3.2 primers (Figure 4). A summary of mean ± SD performance metrics across mutation conditions, stratified by ARTIC version and sequencing error model, is provided for each tool in Supplementary Figure S6. This was due to BWA’s misalignment of reads under the v.5.3.2 primers design, which starts forward primer at position 47, exacerbating the position 46 misalignment issue observed in shotgun data. With HISAT2, the sgRNAdetect’s performance mirrored LeTRS’s limitations, showing reduced performances in datasets with two or three N gene mutations.

LeTRS performance was influenced by both read length and aligner choice. With 300 bp reads (MiSeq), LeTRS achieved good performances across all dataset mutations, comparable to other tools, for both ARTIC v.4 and v.5.3.2. However, with 125 bp reads (HiSeq), its ability to correctly detect sgRNA events dropped in the presence of three N gene mutations, reflecting HISAT2’s difficulty with shorter reads containing multiple mutations.

### 3.4 Analysis on wastewater surveillance datasets

**FIGURE 5:**
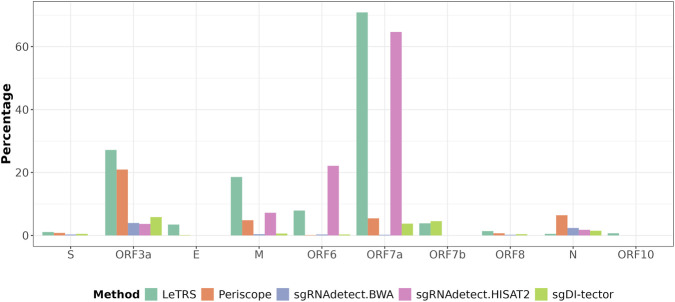
Percentages of samples showing sgRNAs evidence. The plot displays the percentage of samples from the wastewater dataset positive for each sgRNA species. Colours were used to discriminate between tools and mappers.

**FIGURE 6:**
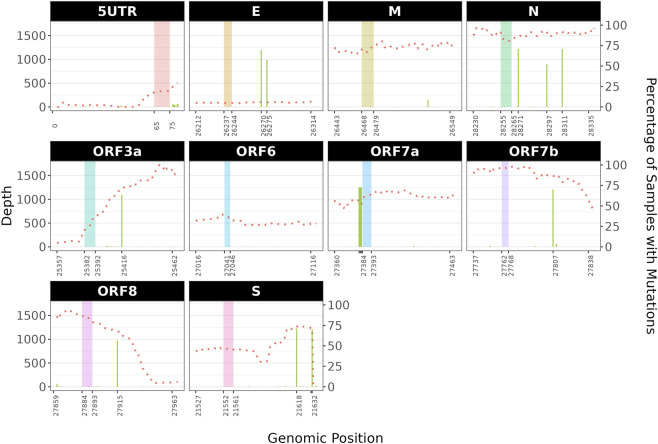
Mutation profiles of the wastewater dataset. For each genomic region of the SARS-CoV-2 genome, we: i. Reported the median depth of coverage (dotted red lines); ii. Highlighted with coloured boxes the TRS-L (in the 5UTR box) and the TRS-B regions and iii. Displayed the percentage of samples with mutations with green barplots. More than 50% of the samples in the wastewater dataset displayed three consecutive mutations in the proximal 5′TRS-B sequence of the ORF7a gene and three additional mutations close to the TRS-B site within the N gene.

To better understand whether the four tools can keep their robustness under real scenarios, we decided to perform a stress test by analysing a dataset composed by ca. 1,000 samples from 29 different wastewater plants, sequenced with Illumina technology (NextSeq instrument, paired-end reads of 74 bp in length), following the ARTIC v.5.3.2 amplicon strategy (project ID: PRJEB65603). Although previous studies using the sgRNAdetect showed low sgRNA presence in some of these samples and seasonal discrepancies in the abundance of sgRNAs (Tang et al., 2024), we specifically selected this dataset to better understand whether our previous *in-silico*-based observations can be successfully transferred on real data, including those from complex sources such as wastewater samples, in which multiple SARS-CoV-2 lineages with heterogeneous frequencies can be present, and that are subject to possible degradation of the RNA. Due to the complexity of the dataset and to the lack of harmonisation in the tools’ output normalisation strategies, we solely focused on qualitative analyses (i.e., identifying which samples are positive for sgRNA events) rather than comparing sgRNA levels. We observed discrepancies in the number of samples positives for sgRNA events according to the tool used, in particular related to sgRNA ORF7a (Figure 5). Additionally, we observed multiple mutations close to the locations of ORF7a and N gene TRS-Bs (Figure 6). Since our *in silico* results highlighted the effects of multiple mutations on the tool’s performances we decided to focus only on sgRNA events for ORF7a and the N genes.

In the N gene, LeTRS was almost completely unable to identify sgRNA events, whereas sgDI-tector and Periscope performed well. Manual inspection of the immediate 5′ region of the N gene revealed three closely spaced mutations (A28271T, T28297C and C28311T). Consistent with our previous observations on *in silico* data, we hypothesise that these impair LeTRS’s ability to detect sgRNA N events. To test this hypothesis, we manually examined two samples that were positive for sgRNA N by LeTRS. In one sample, the sgRNA N event was supported by multiple reads and no mutations were present. In the other sample, only a single supporting read was observed; this read contained all three mutated bases and exhibited low quality (Supplementary Figure S7).

**FIGURE 7:**
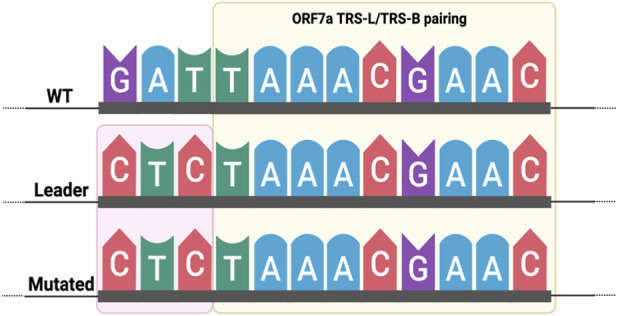
Base-pairing between the SARS-CoV-2 leader TRS (TRS-L) and the ORF7a body TRS (TRS-B). In the wild-type (WT) sequence, a 9-nucleotide stretch including the canonical TRS-B core perfectly complements the TRS-L (highlighted in yellow). The three adjacent mutations (G27382C, A27383T, T27384C), located immediately upstream of the wild-type ORF7a TRS-B (highlighted in red), extend the complementary pairing with the TRS-L from 9 to 12 bp in a mutated SARS-CoV-2 sequence.

We next examined the ORF7a locus to determine the source of the observed discrepancies. Manual inspection revealed a concurrent 3-bases mutation (G27382C, A27383T and T27384C) immediately adjacent to the ORF7a TRS-B start site, which extends the overlapping region between the TRS-L and TRS-B (Figure 7). We hypothesise that these mutations interfere with the normal mapping behaviour of the HISAT2 step of LeTRS, thereby confusing the identification and quantification of the corresponding sgRNA species. To test whether the aligner was driving the LeTRS results, we re-analysed HISAT2-aligned reads with sgRNAdetect, and we observed an increase in the number of positive samples, matching those reported by LeTRS.

## 4 Discussion

This study benchmarked four bioinformatics tools, LeTRS, Periscope, sgDI-tector, and the sgRNAdetect pipeline, for detecting SARS-CoV-2 subgenomic RNAs (sgRNAs), specifically targeting the nucleocapsid (N) gene, using 25 synthetic Illumina datasets and a real-world wastewater dataset. Our publicly available datasets, accessible at European Commission and Joint Research Centre (2026), address a critical gap in standardised benchmarking resources for sgRNA detection tools (Mori et al., 2023). By evaluating the impact of sequencing strategies, mutation profiles, aligner choices, and primer designs, we provide evidence-based guidance for context-specific tool selection, enhancing the reliability of sgRNA quantification for genomic surveillance and diagnostics. Our findings highlight significant performance variability across tools, driven by the screening approach, sequencing strategy and aligner choice, or a combination of them (Table 4). For example, the underperformance of Periscope when dealing with datasets from shotgun sequencing strategy datasets can be attributed to its sequence bait approach, which considers only split reads with at least six nucleotides in the soft-clipped portion of reads (bait sequence: AACCAACTTTCGATCTCTTGTAGATCTGTTCTC). This approach is not effective in detecting reads with partial or mutated transcription regulatory sequences (TRSs), as it may not account for discordant reads supporting sgRNA events. In contrast, sgDI-tector demonstrated superior robustness across all conditions (mean F-score: 0.89 for shotgun, 0.99 for amplicon), owing to its leader-independent detection approach, making it suitable for diverse sequencing scenarios. Its robustness was evident in detecting sgRNA-supporting reads even in mutation-containing datasets. However, its limitation in leveraging paired-end reads suggests potential for improvement in paired-end data integration. SgRNAdetect performed well (mean F-score: 0.85 for shotgun, 0.90 for amplicon with HISAT2), but since this method uses BWA alignments as standard input, it becomes sensitive to transcription regulatory sequence (TRS) mutations and misalignments, particularly with ARTIC v.5.3.2 primers, underscoring the need for mutation-aware workflows. LeTRS showed strong performance in wild-type datasets but faltered with multiple N gene mutations (mean F-score: 0.62 for shotgun with mutations), reflecting HISAT2’s sensitivity to sequence variations. Periscope excelled in amplicon sequencing (mean F-score: 1.0 with BWA) but failed with HISAT2-mapped data due to its reliance on split reads, limiting its versatility in mutation-rich datasets. Our observations (Supplementary Figures S3, S4) on the limitations of HISAT2-based approaches for analysing mutated viral lineages suggest that HISAT2’s sensitivity to mutations in the N gene region compromises its utility in this context.

**TABLE 4:** Comparison of sgRNA detection tools. Summary of strengths, limitations, and peculiarities of the bioinformatics tools (sgDI-tector, Periscope, LeTRS, and sgRNAdetect) used for SARS-CoV-2 subgenomic RNA (sgRNA) detection.

Attribute	LeTRS	sgDI-tector	Periscope	sgRNAdetect
Strengths	Identifies both c- and nc-sgRNA; flexible input (FASTQ or BAM)	No TRS knowledge required; detects nc-sgRNA effectively	Handles raw reads; optimised for ARTIC protocols; fast for paired-end data	Leverages paired-end data for precise sgRNA calling
Limitations	Complex preprocessing	Less suited for paired-end data; requires ORF reference for annotation	Limited to TRS-dependent sgRNA	Excludes reads not mapping to the last third of the virus genome
Commonalities	Adaptable to other viruses; tested on SARS-CoV-2 amplicon ARTIC data	Adaptable to other viruses; tested on SARS-CoV-2 amplicon ARTIC data	Adaptable to other viruses; tested on SARS-CoV-2 amplicon ARTIC data	Tested on SARS-CoV-2 amplicon ARTIC data
Peculiarities	Perl-based; distinguishes leader-independent nc-sgRNA junctions	Uses DI-tector for defective viral genomes; annotates sgRNAs with user-provided ORF reference	Designed for nanopore ARTIC protocols; no preprocessing needed	Custom pipeline; requires specific 5′ UTR–last third of virus genome mate pairing for sgRNA detection

Aligner choice emerged as a pivotal factor influencing tool performance. BWA’s robustness to mutations makes it preferable for analysing emerging viral variants, while HISAT2’s splice-aware mapping enhances detection in wild-type datasets but struggles with multiple mutations, particularly with shorter reads (125 bp). Amplicon sequencing outperformed shotgun sequencing due to its targeted nature, but primer design, exemplified by ARTIC v.5.3.2’s amplicon 1 starting at position 47, can exacerbate aligner-related challenges, particularly for BWA-based pipelines. Indeed, when using BWA, reads starting at position 46 were excluded from consideration, leading to underestimation of sgRNA counts. This observation emphasises the critical role of aligner selection in workflow design and the need for careful evaluation of each pipeline component to ensure robust analysis. In addition, it had been shown that different ARTIC primer schemes can generate inconsistent PCR artefacts for the same sample, materialised into ambiguous base calls, that would exist when using one primer scheme but not with another, in particular when mutations within primer sites occur (Ellegaard et al., 2025). The use of the most suited primer design for the studied samples is therefore critical to mitigate amplicon related issues. These insights guide workflow optimisation: for clinical applications requiring mutation robustness, sgDI-tector or BWA-based pipelines are optimal, while Periscope is well-suited for amplicon-based epidemiological surveillance.

Analysis of the wastewater dataset (PRJEB65603) validated our synthetic findings in a real-world context, highlighting challenges in detecting low-abundance sgRNAs in samples with diverse SARS-CoV-2 lineages. Discrepancies in detecting sgRNA ORF7a and N events, particularly with LeTRS, were linked to mutations near TRS regions, reinforcing the need for mutation-robust tools in complex samples. Our datasets and benchmarking framework mitigate quantification errors, supporting more accurate sgRNA-based diagnostics and surveillance. The standardised datasets generated in this study are a reusable resource for the research community, enabling the development and validation of new sgRNA detection tools. By simulating diverse sequencing conditions, they facilitate rigorous benchmarking, addressing the scarcity of such resources noted by Mori et al. (2023) (Di Gioacchino et al., 2022). These datasets can accelerate advancements in sgRNA-based diagnostics and extend to other coronaviruses or RNA viruses with similar transcription mechanisms.

Our study has limitations that warrant consideration. First, reliance on Illumina-based synthetic datasets may not fully capture the complexities of clinical samples, such as variable viral loads or host RNA interference. Second, the focus on sgRNA N limits generalisability to other sgRNAs, such as ORF7a, which showed distinct mutation-related challenges in wastewater analyses. Third, the study did not explore long-read sequencing technologies (e.g., Oxford Nanopore), which could offer advantages in resolving complex sgRNA junctions. Future research should validate these findings with clinical samples across diverse viral loads and lineages. Extending the benchmarking framework to long-read technologies and other sgRNAs or viruses would enhance its applicability. Integrating machine learning approaches, such as those for improved junction detection, could address limitations like Periscope’s split-read dependency or sgDI-tector’s single-end read constraint. Finally, we believe that community-driven standards, inspired by initiatives like the Global Alliance for Genomics and Health (GA4GH) (Rehm et al., 2021), are essential to ensure cross-study comparability and reproducibility in sgRNA detection workflows.

This study underscores the critical role of systematic benchmarking in optimising bioinformatics workflows for sgRNA detection. By elucidating the impact of sequencing strategies, aligners, and mutation profiles, we provide actionable insights for tool selection in SARS-CoV-2 surveillance and diagnostics. Our publicly available datasets empower researchers to refine existing tools and develop new ones, advancing the reliability of sgRNA-based applications. While clinical validation of sgRNA’s diagnostic potential remains a priority, our findings and resources lay a robust foundation for genomic surveillance of COVID-19 and might be translated to different targets in case of future pandemics.

The implications of our study extend to public health policy and practice. By demonstrating the impact of systematic benchmarking on bioinformatics workflow reliability for sgRNA detection, our findings support the development of evidence-based guidelines for genomic surveillance tools in clinical and epidemiological settings. Standardised benchmarking frameworks, aligned with global initiatives such as the WHO’s Genomic Surveillance Strategy and One Health principles, can promote cross-study comparability, reproducibility, and equitable data sharing. This, in turn, strengthens real-time monitoring of pathogen transmission and evolution, informing targeted interventions and global health security. As the research community prepares for future infectious disease threats, rigorous tool evaluation and shared benchmarking resources, such as those provided here, will be essential to advance reliable genomic surveillance consistent with One Health principles.

## Data Availability

The raw data generated for this study can be found at the Joint Research Centre Data Catalogue (https://doi.org/10.2905/JRC.1RYH4D0). The wastewater dataset analysed in this study can be found at the European Nucleotide Archive (ENA) under the Project ID: PRJEB65603.
