# Supplementary material for: How benchmarking of bioinformatics tools is essential for informed workflow selection: a case study on SARS-CoV-2 subgenomic RNA detection
Source: Front Bioinform. 2026 Apr 22;6:1803237. doi: 10.3389/fbinf.2026.1803237 (PMC13144104; doi:10.3389/fbinf.2026.1803237)
Supplement: Supplementary file 1 [file Supplementaryfile1.docx]

***Supplementary Material***

**Supplementary Figures and Tables**

**Supplementary Tables**

| Tool | ID | TP | FN | FP | TN | Precision | Recall | Accuracy | Fscore |
| --- | --- | --- | --- | --- | --- | --- | --- | --- | --- |
| LeTRS | WT | 56 | 18 | 0 | 26 | 1 | 0.76 | 0.82 | 0.86 |
| LeTRS | TRS-B | 33 | 41 | 0 | 26 | 1 | 0.45 | 0.59 | 0.62 |
| LeTRS | ORF_N | 41 | 33 | 0 | 26 | 1 | 0.55 | 0.67 | 0.71 |
| LeTRS | 2_ORF_N | 20 | 54 | 0 | 26 | 1 | 0.27 | 0.46 | 0.43 |
| LeTRS | 3_ORF_N | 25 | 49 | 0 | 26 | 1 | 0.34 | 0.51 | 0.51 |
| Periscope | WT | 26 | 48 | 0 | 26 | 1 | 0.35 | 0.52 | 0.52 |
| Periscope | TRS-B | 26 | 48 | 0 | 26 | 1 | 0.35 | 0.52 | 0.52 |
| Periscope | ORF_N | 24 | 50 | 0 | 26 | 1 | 0.32 | 0.50 | 0.49 |
| Periscope | 2_ORF_N | 24 | 50 | 0 | 26 | 1 | 0.32 | 0.50 | 0.49 |
| Periscope | 3_ORF_N | 22 | 52 | 0 | 26 | 1 | 0.30 | 0.48 | 0.46 |
| sgDI-tector | WT | 59 | 15 | 0 | 26 | 1 | 0.80 | 0.85 | 0.89 |
| sgDI-tector | TRS-B | 58 | 16 | 0 | 26 | 1 | 0.78 | 0.84 | 0.88 |
| sgDI-tector | ORF_N | 59 | 15 | 0 | 26 | 1 | 0.80 | 0.85 | 0.89 |
| sgDI-tector | 2_ORF_N | 59 | 15 | 0 | 26 | 1 | 0.80 | 0.85 | 0.89 |
| sgDI-tector | 3_ORF_N | 59 | 15 | 0 | 26 | 1 | 0.80 | 0.85 | 0.89 |
| sgRNAdetect | WT | 56 | 18 | 0 | 26 | 1 | 0.76 | 0.82 | 0.86 |
| sgRNAdetect | TRS-B | 51 | 23 | 0 | 26 | 1 | 0.69 | 0.77 | 0.82 |
| sgRNAdetect | ORF_N | 56 | 18 | 0 | 26 | 1 | 0.76 | 0.82 | 0.86 |
| sgRNAdetect | 2_ORF_N | 56 | 18 | 0 | 26 | 1 | 0.76 | 0.82 | 0.86 |
| sgRNAdetect | 3_ORF_N | 56 | 18 | 0 | 26 | 1 | 0.76 | 0.82 | 0.86 |

**Supplementary Table 1: Shotgun Performance Scores.** Performance metrics (True Positives, False Negatives, False Positives, True Negatives, Precision, Recall, Accuracy, Fscore) for LeTRS, Periscope, sgDI-tector, and sgRNAdetect across five shotgun datasets (wild-type, TRS-B mutation, and 1–3 N gene mutations). Metrics were calculated based on manual inspection of BAM files using IGV. Precision, Recall, Accuracy and Fscore were evaluated using the following formulas: Precision = TP / (TP + FP); Recall = TP / (TP + FN); Accuracy = (TP + TN) / (TP + TN + FP + FN); Fscore = 2 * (Precision * Recall) / (Precision + Recall).

| Tool | Dataset | Version | Technology | Mapper | TP | TN | FP | FN | Precision | Recall | Accuracy | Fscore |
| --- | --- | --- | --- | --- | --- | --- | --- | --- | --- | --- | --- | --- |
| LeTRS | 2_ORF_N | 4 | hiseq | HiSat2 | 47 | 0 | 0 | 3 | 1.00 | 0.94 | 0.94 | 0.97 |
| LeTRS | 2_ORF_N | 4 | miseq | HiSat2 | 50 | 0 | 0 | 0 | 1.00 | 1.00 | 1.00 | 1.00 |
| LeTRS | 2_ORF_N | 5.3.2 | hiseq | HiSat2 | 45 | 0 | 0 | 5 | 1.00 | 0.90 | 0.90 | 0.95 |
| LeTRS | 2_ORF_N | 5.3.2 | miseq | HiSat2 | 47 | 0 | 0 | 3 | 1.00 | 0.94 | 0.94 | 0.97 |
| LeTRS | 3_ORF_N | 4 | hiseq | HiSat2 | 4 | 0 | 0 | 46 | 1.00 | 0.08 | 0.08 | 0.15 |
| LeTRS | 3_ORF_N | 4 | miseq | HiSat2 | 48 | 0 | 0 | 2 | 1.00 | 0.96 | 0.96 | 0.98 |
| LeTRS | 3_ORF_N | 5.3.2 | hiseq | HiSat2 | 4 | 0 | 0 | 46 | 1.00 | 0.08 | 0.08 | 0.15 |
| LeTRS | 3_ORF_N | 5.3.2 | miseq | HiSat2 | 50 | 0 | 0 | 0 | 1.00 | 1.00 | 1.00 | 1.00 |
| LeTRS | ORF_N | 4 | hiseq | HiSat2 | 48 | 0 | 0 | 2 | 1.00 | 0.96 | 0.96 | 0.98 |
| LeTRS | ORF_N | 4 | miseq | HiSat2 | 49 | 0 | 0 | 1 | 1.00 | 0.98 | 0.98 | 0.99 |
| LeTRS | ORF_N | 5.3.2 | hiseq | HiSat2 | 50 | 0 | 0 | 0 | 1.00 | 1.00 | 1.00 | 1.00 |
| LeTRS | ORF_N | 5.3.2 | miseq | HiSat2 | 50 | 0 | 0 | 0 | 1.00 | 1.00 | 1.00 | 1.00 |
| LeTRS | TRS-B | 4 | hiseq | HiSat2 | 50 | 0 | 0 | 0 | 1.00 | 1.00 | 1.00 | 1.00 |
| LeTRS | TRS-B | 4 | miseq | HiSat2 | 50 | 0 | 0 | 0 | 1.00 | 1.00 | 1.00 | 1.00 |
| LeTRS | TRS-B | 5.3.2 | hiseq | HiSat2 | 50 | 0 | 0 | 0 | 1.00 | 1.00 | 1.00 | 1.00 |
| LeTRS | TRS-B | 5.3.2 | miseq | HiSat2 | 47 | 0 | 0 | 3 | 1.00 | 0.94 | 0.94 | 0.97 |
| LeTRS | WT | 4 | hiseq | HiSat2 | 50 | 0 | 0 | 0 | 1.00 | 1.00 | 1.00 | 1.00 |
| LeTRS | WT | 4 | miseq | HiSat2 | 50 | 0 | 0 | 0 | 1.00 | 1.00 | 1.00 | 1.00 |
| LeTRS | WT | 5.3.2 | hiseq | HiSat2 | 50 | 0 | 0 | 0 | 1.00 | 1.00 | 1.00 | 1.00 |
| LeTRS | WT | 5.3.2 | miseq | HiSat2 | 48 | 0 | 0 | 2 | 1.00 | 0.96 | 0.96 | 0.98 |
| Periscope | 2_ORF_N | 4 | hiseq | Bwa | 50 | 0 | 0 | 0 | 1.00 | 1.00 | 1.00 | 1.00 |
| Periscope | 2_ORF_N | 4 | hiseq | HiSat2 | 0 | 0 | 0 | 50 | 0.00 | 0.00 | 0.00 | 0.00 |
| Periscope | 2_ORF_N | 4 | miseq | Bwa | 50 | 0 | 0 | 0 | 1.00 | 1.00 | 1.00 | 1.00 |
| Periscope | 2_ORF_N | 4 | miseq | HiSat2 | 0 | 0 | 0 | 50 | 0.00 | 0.00 | 0.00 | 0.00 |
| Periscope | 2_ORF_N | 5.3.2 | hiseq | Bwa | 49 | 0 | 0 | 1 | 1.00 | 0.98 | 0.98 | 0.99 |
| Periscope | 2_ORF_N | 5.3.2 | hiseq | HiSat2 | 0 | 0 | 0 | 50 | 0.00 | 0.00 | 0.00 | 0.00 |
| Periscope | 2_ORF_N | 5.3.2 | miseq | Bwa | 50 | 0 | 0 | 0 | 1.00 | 1.00 | 1.00 | 1.00 |
| Periscope | 2_ORF_N | 5.3.2 | miseq | HiSat2 | 0 | 0 | 0 | 50 | 0.00 | 0.00 | 0.00 | 0.00 |
| Periscope | 3_ORF_N | 4 | hiseq | Bwa | 50 | 0 | 0 | 0 | 1.00 | 1.00 | 1.00 | 1.00 |
| Periscope | 3_ORF_N | 4 | hiseq | HiSat2 | 0 | 0 | 0 | 50 | 0.00 | 0.00 | 0.00 | 0.00 |
| Periscope | 3_ORF_N | 4 | miseq | Bwa | 50 | 0 | 0 | 0 | 1.00 | 1.00 | 1.00 | 1.00 |
| Periscope | 3_ORF_N | 4 | miseq | HiSat2 | 0 | 0 | 0 | 50 | 0.00 | 0.00 | 0.00 | 0.00 |
| Periscope | 3_ORF_N | 5.3.2 | hiseq | Bwa | 50 | 0 | 0 | 0 | 1.00 | 1.00 | 1.00 | 1.00 |
| Periscope | 3_ORF_N | 5.3.2 | hiseq | HiSat2 | 0 | 0 | 0 | 50 | 0.00 | 0.00 | 0.00 | 0.00 |
| Periscope | 3_ORF_N | 5.3.2 | miseq | Bwa | 50 | 0 | 0 | 0 | 1.00 | 1.00 | 1.00 | 1.00 |
| Periscope | 3_ORF_N | 5.3.2 | miseq | HiSat2 | 0 | 0 | 0 | 50 | 0.00 | 0.00 | 0.00 | 0.00 |
| Periscope | ORF_N | 4 | hiseq | Bwa | 50 | 0 | 0 | 0 | 1.00 | 1.00 | 1.00 | 1.00 |
| Periscope | ORF_N | 4 | hiseq | HiSat2 | 0 | 0 | 0 | 50 | 0.00 | 0.00 | 0.00 | 0.00 |
| Periscope | ORF_N | 4 | miseq | Bwa | 50 | 0 | 0 | 0 | 1.00 | 1.00 | 1.00 | 1.00 |
| Periscope | ORF_N | 4 | miseq | HiSat2 | 0 | 0 | 0 | 50 | 0.00 | 0.00 | 0.00 | 0.00 |
| Periscope | ORF_N | 5.3.2 | hiseq | Bwa | 50 | 0 | 0 | 0 | 1.00 | 1.00 | 1.00 | 1.00 |
| Periscope | ORF_N | 5.3.2 | hiseq | HiSat2 | 0 | 0 | 0 | 50 | 0.00 | 0.00 | 0.00 | 0.00 |
| Periscope | ORF_N | 5.3.2 | miseq | Bwa | 50 | 0 | 0 | 0 | 1.00 | 1.00 | 1.00 | 1.00 |
| Periscope | ORF_N | 5.3.2 | miseq | HiSat2 | 0 | 0 | 0 | 50 | 0.00 | 0.00 | 0.00 | 0.00 |
| Periscope | TRS-B | 4 | hiseq | Bwa | 50 | 0 | 0 | 0 | 1.00 | 1.00 | 1.00 | 1.00 |
| Periscope | TRS-B | 4 | hiseq | HiSat2 | 0 | 0 | 0 | 50 | 0.00 | 0.00 | 0.00 | 0.00 |
| Periscope | TRS-B | 4 | miseq | Bwa | 50 | 0 | 0 | 0 | 1.00 | 1.00 | 1.00 | 1.00 |
| Periscope | TRS-B | 4 | miseq | HiSat2 | 0 | 0 | 0 | 50 | 0.00 | 0.00 | 0.00 | 0.00 |
| Periscope | TRS-B | 5.3.2 | hiseq | Bwa | 50 | 0 | 0 | 0 | 1.00 | 1.00 | 1.00 | 1.00 |
| Periscope | TRS-B | 5.3.2 | hiseq | HiSat2 | 0 | 0 | 0 | 50 | 0.00 | 0.00 | 0.00 | 0.00 |
| Periscope | TRS-B | 5.3.2 | miseq | Bwa | 49 | 0 | 0 | 1 | 1.00 | 0.98 | 0.98 | 0.99 |
| Periscope | TRS-B | 5.3.2 | miseq | HiSat2 | 0 | 0 | 0 | 50 | 0.00 | 0.00 | 0.00 | 0.00 |
| Periscope | WT | 4 | hiseq | Bwa | 50 | 0 | 0 | 0 | 1.00 | 1.00 | 1.00 | 1.00 |
| Periscope | WT | 4 | hiseq | HiSat2 | 0 | 0 | 0 | 50 | 0.00 | 0.00 | 0.00 | 0.00 |
| Periscope | WT | 4 | miseq | Bwa | 50 | 0 | 0 | 0 | 1.00 | 1.00 | 1.00 | 1.00 |
| Periscope | WT | 4 | miseq | HiSat2 | 0 | 0 | 0 | 50 | 0.00 | 0.00 | 0.00 | 0.00 |
| Periscope | WT | 5.3.2 | hiseq | Bwa | 50 | 0 | 0 | 0 | 1.00 | 1.00 | 1.00 | 1.00 |
| Periscope | WT | 5.3.2 | hiseq | HiSat2 | 0 | 0 | 0 | 50 | 0.00 | 0.00 | 0.00 | 0.00 |
| Periscope | WT | 5.3.2 | miseq | Bwa | 50 | 0 | 0 | 0 | 1.00 | 1.00 | 1.00 | 1.00 |
| Periscope | WT | 5.3.2 | miseq | HiSat2 | 0 | 0 | 0 | 50 | 0.00 | 0.00 | 0.00 | 0.00 |
| sgDI-tector | 2_ORF_N | 4 | hiseq | Bwa | 50 | 0 | 0 | 0 | 1.00 | 1.00 | 1.00 | 1.00 |
| sgDI-tector | 2_ORF_N | 4 | miseq | Bwa | 49 | 0 | 0 | 1 | 1.00 | 0.98 | 0.98 | 0.99 |
| sgDI-tector | 2_ORF_N | 5.3.2 | hiseq | Bwa | 49 | 0 | 0 | 1 | 1.00 | 0.98 | 0.98 | 0.99 |
| sgDI-tector | 2_ORF_N | 5.3.2 | miseq | Bwa | 50 | 0 | 0 | 0 | 1.00 | 1.00 | 1.00 | 1.00 |
| sgDI-tector | 3_ORF_N | 4 | hiseq | Bwa | 49 | 0 | 0 | 1 | 1.00 | 0.98 | 0.98 | 0.99 |
| sgDI-tector | 3_ORF_N | 4 | miseq | Bwa | 49 | 0 | 0 | 1 | 1.00 | 0.98 | 0.98 | 0.99 |
| sgDI-tector | 3_ORF_N | 5.3.2 | hiseq | Bwa | 49 | 0 | 0 | 1 | 1.00 | 0.98 | 0.98 | 0.99 |
| sgDI-tector | 3_ORF_N | 5.3.2 | miseq | Bwa | 48 | 0 | 0 | 2 | 1.00 | 0.96 | 0.96 | 0.98 |
| sgDI-tector | ORF_N | 4 | hiseq | Bwa | 49 | 0 | 0 | 1 | 1.00 | 0.98 | 0.98 | 0.99 |
| sgDI-tector | ORF_N | 4 | miseq | Bwa | 50 | 0 | 0 | 0 | 1.00 | 1.00 | 1.00 | 1.00 |
| sgDI-tector | ORF_N | 5.3.2 | hiseq | Bwa | 50 | 0 | 0 | 0 | 1.00 | 1.00 | 1.00 | 1.00 |
| sgDI-tector | ORF_N | 5.3.2 | miseq | Bwa | 50 | 0 | 0 | 0 | 1.00 | 1.00 | 1.00 | 1.00 |
| sgDI-tector | TRS-B | 4 | hiseq | Bwa | 50 | 0 | 0 | 0 | 1.00 | 1.00 | 1.00 | 1.00 |
| sgDI-tector | TRS-B | 4 | miseq | Bwa | 50 | 0 | 0 | 0 | 1.00 | 1.00 | 1.00 | 1.00 |
| sgDI-tector | TRS-B | 5.3.2 | hiseq | Bwa | 50 | 0 | 0 | 0 | 1.00 | 1.00 | 1.00 | 1.00 |
| sgDI-tector | TRS-B | 5.3.2 | miseq | Bwa | 50 | 0 | 0 | 0 | 1.00 | 1.00 | 1.00 | 1.00 |
| sgDI-tector | WT | 4 | hiseq | Bwa | 50 | 0 | 0 | 0 | 1.00 | 1.00 | 1.00 | 1.00 |
| sgDI-tector | WT | 4 | miseq | Bwa | 50 | 0 | 0 | 0 | 1.00 | 1.00 | 1.00 | 1.00 |
| sgDI-tector | WT | 5.3.2 | hiseq | Bwa | 50 | 0 | 0 | 0 | 1.00 | 1.00 | 1.00 | 1.00 |
| sgDI-tector | WT | 5.3.2 | miseq | Bwa | 49 | 0 | 0 | 1 | 1.00 | 0.98 | 0.98 | 0.99 |
| sgRNAdetect | 2_ORF_N | 4 | hiseq | Bwa | 50 | 0 | 0 | 0 | 1.00 | 1.00 | 1.00 | 1.00 |
| sgRNAdetect | 2_ORF_N | 4 | hiseq | HiSat2 | 47 | 0 | 0 | 3 | 1.00 | 0.94 | 0.94 | 0.97 |
| sgRNAdetect | 2_ORF_N | 4 | miseq | Bwa | 50 | 0 | 0 | 0 | 1.00 | 1.00 | 1.00 | 1.00 |
| sgRNAdetect | 2_ORF_N | 4 | miseq | HiSat2 | 50 | 0 | 0 | 0 | 1.00 | 1.00 | 1.00 | 1.00 |
| sgRNAdetect | 2_ORF_N | 5.3.2 | hiseq | Bwa | 0 | 0 | 0 | 0 | 0.00 | 0.00 | 0.00 | 0.00 |
| sgRNAdetect | 2_ORF_N | 5.3.2 | hiseq | HiSat2 | 46 | 0 | 0 | 4 | 1.00 | 0.92 | 0.92 | 0.96 |
| sgRNAdetect | 2_ORF_N | 5.3.2 | miseq | Bwa | 0 | 0 | 0 | 0 | 0.00 | 0.00 | 0.00 | 0.00 |
| sgRNAdetect | 2_ORF_N | 5.3.2 | miseq | HiSat2 | 47 | 0 | 0 | 3 | 1.00 | 0.94 | 0.94 | 0.97 |
| sgRNAdetect | 3_ORF_N | 4 | hiseq | Bwa | 49 | 0 | 0 | 1 | 1.00 | 0.98 | 0.98 | 0.99 |
| sgRNAdetect | 3_ORF_N | 4 | hiseq | HiSat2 | 4 | 0 | 0 | 46 | 1.00 | 0.08 | 0.08 | 0.15 |
| sgRNAdetect | 3_ORF_N | 4 | miseq | Bwa | 50 | 0 | 0 | 0 | 1.00 | 1.00 | 1.00 | 1.00 |
| sgRNAdetect | 3_ORF_N | 4 | miseq | HiSat2 | 46 | 0 | 0 | 4 | 1.00 | 0.92 | 0.92 | 0.96 |
| sgRNAdetect | 3_ORF_N | 5.3.2 | hiseq | Bwa | 0 | 0 | 0 | 0 | 0.00 | 0.00 | 0.00 | 0.00 |
| sgRNAdetect | 3_ORF_N | 5.3.2 | hiseq | HiSat2 | 4 | 0 | 0 | 46 | 1.00 | 0.08 | 0.08 | 0.15 |
| sgRNAdetect | 3_ORF_N | 5.3.2 | miseq | Bwa | 0 | 0 | 0 | 0 | 0.00 | 0.00 | 0.00 | 0.00 |
| sgRNAdetect | 3_ORF_N | 5.3.2 | miseq | HiSat2 | 50 | 0 | 0 | 0 | 1.00 | 1.00 | 1.00 | 1.00 |
| sgRNAdetect | ORF_N | 4 | hiseq | Bwa | 50 | 0 | 0 | 0 | 1.00 | 1.00 | 1.00 | 1.00 |
| sgRNAdetect | ORF_N | 4 | hiseq | HiSat2 | 48 | 0 | 0 | 2 | 1.00 | 0.96 | 0.96 | 0.98 |
| sgRNAdetect | ORF_N | 4 | miseq | Bwa | 50 | 0 | 0 | 0 | 1.00 | 1.00 | 1.00 | 1.00 |
| sgRNAdetect | ORF_N | 4 | miseq | HiSat2 | 49 | 0 | 0 | 1 | 1.00 | 0.98 | 0.98 | 0.99 |
| sgRNAdetect | ORF_N | 5.3.2 | hiseq | Bwa | 0 | 0 | 0 | 0 | 0.00 | 0.00 | 0.00 | 0.00 |
| sgRNAdetect | ORF_N | 5.3.2 | hiseq | HiSat2 | 50 | 0 | 0 | 0 | 1.00 | 1.00 | 1.00 | 1.00 |
| sgRNAdetect | ORF_N | 5.3.2 | miseq | Bwa | 0 | 0 | 0 | 0 | 0.00 | 0.00 | 0.00 | 0.00 |
| sgRNAdetect | ORF_N | 5.3.2 | miseq | HiSat2 | 50 | 0 | 0 | 0 | 1.00 | 1.00 | 1.00 | 1.00 |
| sgRNAdetect | TRS-B | 4 | hiseq | Bwa | 50 | 0 | 0 | 0 | 1.00 | 1.00 | 1.00 | 1.00 |
| sgRNAdetect | TRS-B | 4 | hiseq | HiSat2 | 50 | 0 | 0 | 0 | 1.00 | 1.00 | 1.00 | 1.00 |
| sgRNAdetect | TRS-B | 4 | miseq | Bwa | 48 | 0 | 0 | 2 | 1.00 | 0.96 | 0.96 | 0.98 |
| sgRNAdetect | TRS-B | 4 | miseq | HiSat2 | 50 | 0 | 0 | 0 | 1.00 | 1.00 | 1.00 | 1.00 |
| sgRNAdetect | TRS-B | 5.3.2 | hiseq | Bwa | 0 | 0 | 0 | 0 | 0.00 | 0.00 | 0.00 | 0.00 |
| sgRNAdetect | TRS-B | 5.3.2 | hiseq | HiSat2 | 50 | 0 | 0 | 0 | 1.00 | 1.00 | 1.00 | 1.00 |
| sgRNAdetect | TRS-B | 5.3.2 | miseq | Bwa | 0 | 0 | 0 | 0 | 0.00 | 0.00 | 0.00 | 0.00 |
| sgRNAdetect | TRS-B | 5.3.2 | miseq | HiSat2 | 47 | 0 | 0 | 3 | 1.00 | 0.94 | 0.94 | 0.97 |
| sgRNAdetect | WT | 4 | hiseq | Bwa | 50 | 0 | 0 | 0 | 1.00 | 1.00 | 1.00 | 1.00 |
| sgRNAdetect | WT | 4 | hiseq | HiSat2 | 50 | 0 | 0 | 0 | 1.00 | 1.00 | 1.00 | 1.00 |
| sgRNAdetect | WT | 4 | miseq | Bwa | 50 | 0 | 0 | 0 | 1.00 | 1.00 | 1.00 | 1.00 |
| sgRNAdetect | WT | 4 | miseq | HiSat2 | 50 | 0 | 0 | 0 | 1.00 | 1.00 | 1.00 | 1.00 |
| sgRNAdetect | WT | 5.3.2 | hiseq | Bwa | 0 | 0 | 0 | 0 | 0.00 | 0.00 | 0.00 | 0.00 |
| sgRNAdetect | WT | 5.3.2 | hiseq | HiSat2 | 50 | 0 | 0 | 0 | 1.00 | 1.00 | 1.00 | 1.00 |
| sgRNAdetect | WT | 5.3.2 | miseq | Bwa | 0 | 0 | 0 | 0 | 0.00 | 0.00 | 0.00 | 0.00 |
| sgRNAdetect | WT | 5.3.2 | miseq | HiSat2 | 48 | 0 | 0 | 2 | 1.00 | 0.96 | 0.96 | 0.98 |

**Supplementary Table 2: Amplicon Performance Scores.** Performance metrics (True Positives, False Negatives, False Positives, True Negatives, Precision, Recall, Accuracy, F-score) for LeTRS, Periscope, sgDI-tector, and sgRNAdetect across 20 amplicon datasets, varying by ARTIC version (v.4 vs. v.5.3.2), technology (HiSeq vs. MiSeq), mapper (BWA vs. HISAT2), and mutation profiles. Metrics were derived from BAM file inspections using IGV. Precision, Recall, Accuracy and Fscore were evaluated using the following formulas: Precision = TP / (TP + FP); Recall = TP / (TP + FN); Accuracy = (TP + TN) / (TP + TN + FP + FN); Fscore = 2 * (Precision * Recall) / (Precision + Recall).

**Supplementary Figures**


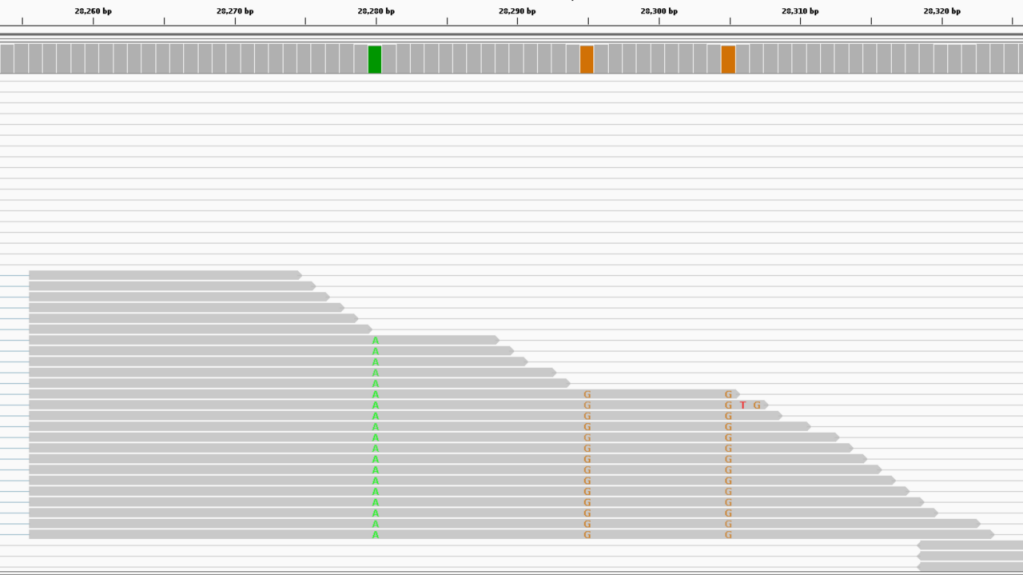


**Supplementary Figure 1: HISAT2 Read Mapping to N Locus.** IGV visualisation of reduced read mapping to the N gene locus by HISAT2 in datasets with multiple N gene mutations. Reads with fewer than 10 nucleotides downstream of mutations were often unmapped, impacting LeTRS performance in detecting sgRNA events.


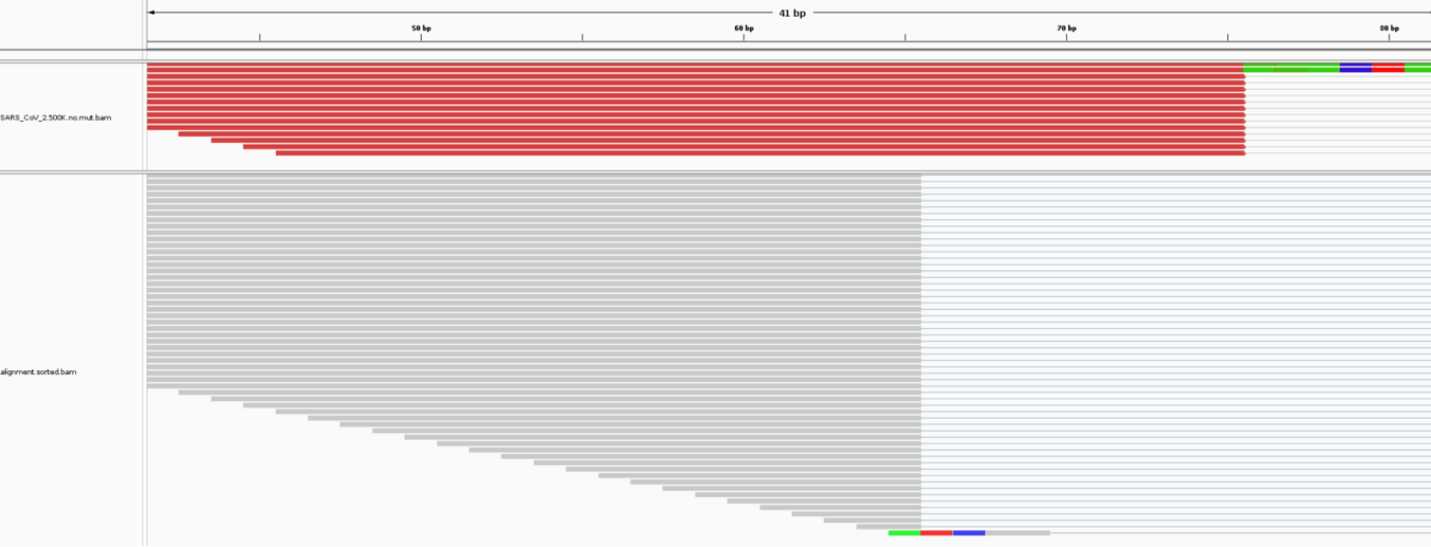


**Supplementary Figure 2: Comparison of BWA and HISAT2 Read Mapping.** Alignment profiles showing BWA correctly mapping reads up to position 46 and HISAT2 accurately mapping reads to the 5’ UTR and TRS-L regions. Together with Supplementary Figure 1, this screenshot illustrates HISAT2’s splice-aware advantage for sgRNA detection but its sensitivity to N gene mutations.


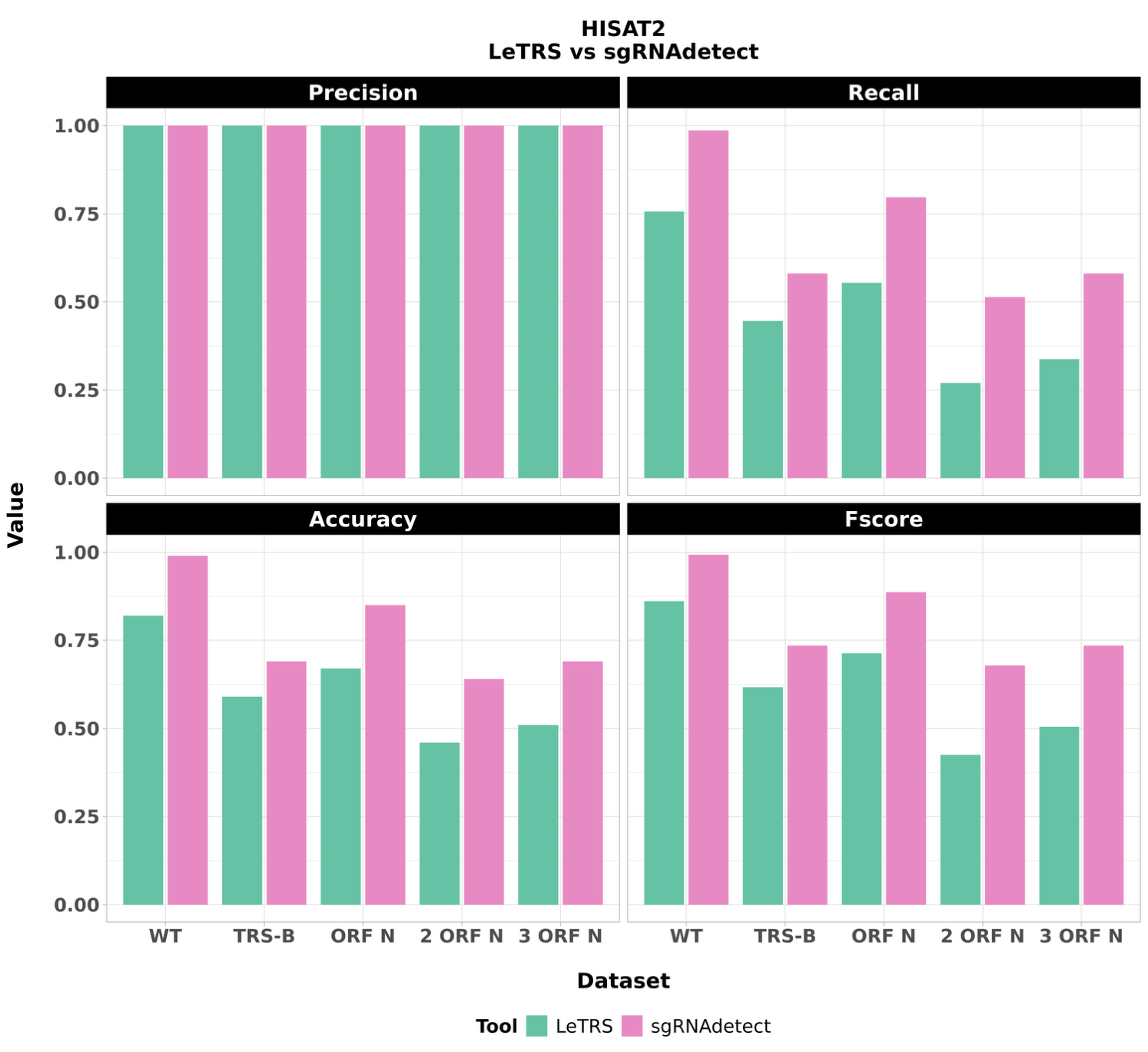


**Supplementary Figure 3: Performances of HISAT2-based tools.** We compared the performances of LeTRS and sgRNAdetect based on HISAT2 alignment. We observed that the HISAT2 alignments negatively affected the performances of the two pipelines when mutations are present within the TRS-B and N gene regions. However, while sgRNAdetect was not natively designed to use HISAT2 aligned data, it showed better performances with respect to LeTRS across all tested conditions.


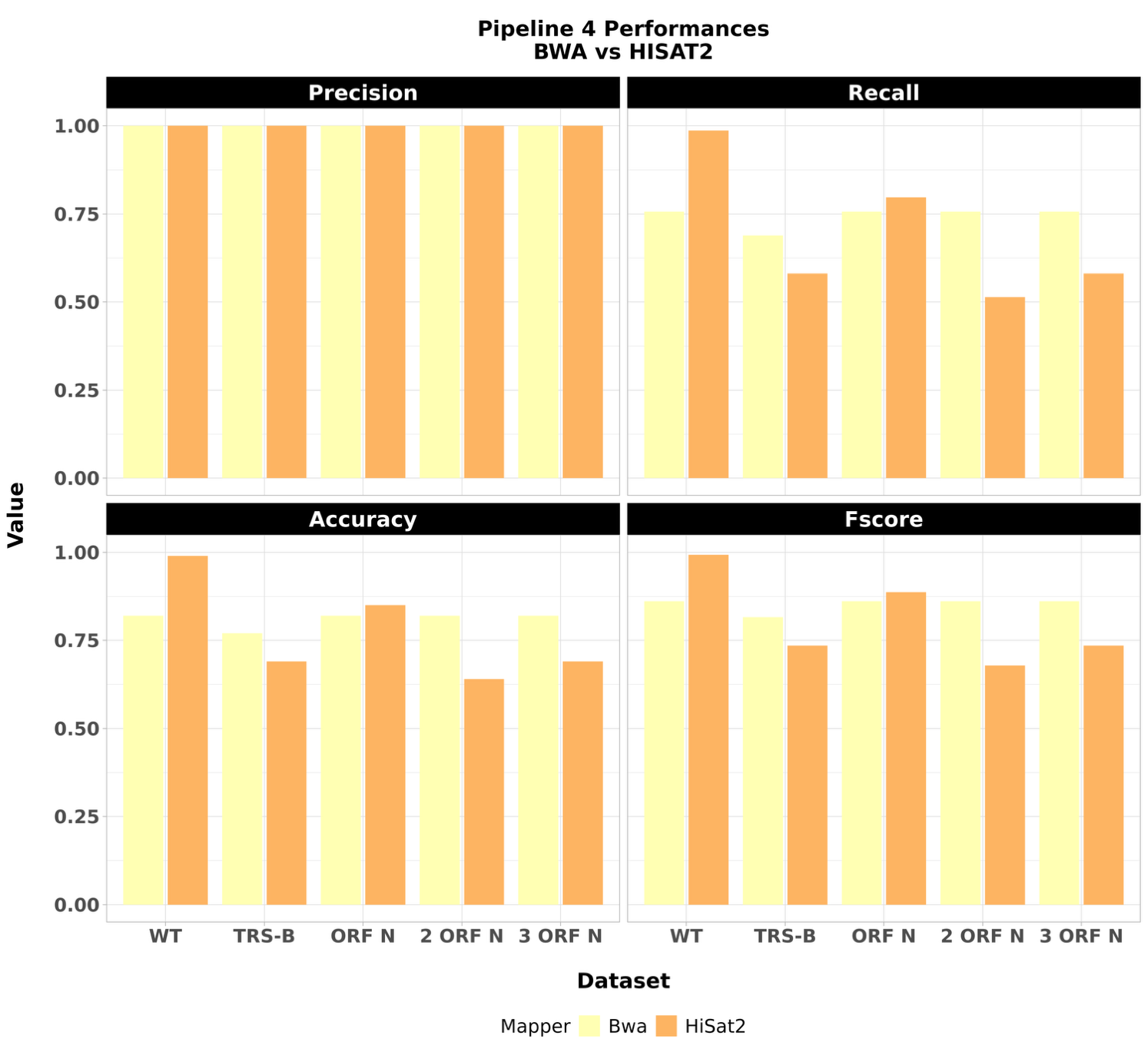


**Supplementary Figure 4: SgRNAdetect performances on whole genome *in-silico* data depending on the chosen aligner.** We evaluated the performances for sgRNAdetect using both BWA and HISAT2 during the alignment step for whole genome *in-silico* data. While we observed increased performances for the WT dataset using HISAT2 due to an improved mapping at the 5’ UTR region, we noticed how HISAT2 generate a decrease in Recall, Accuracy and Fscore when multiple mutations are present with respect to BWA, in line with our previous observations.

**
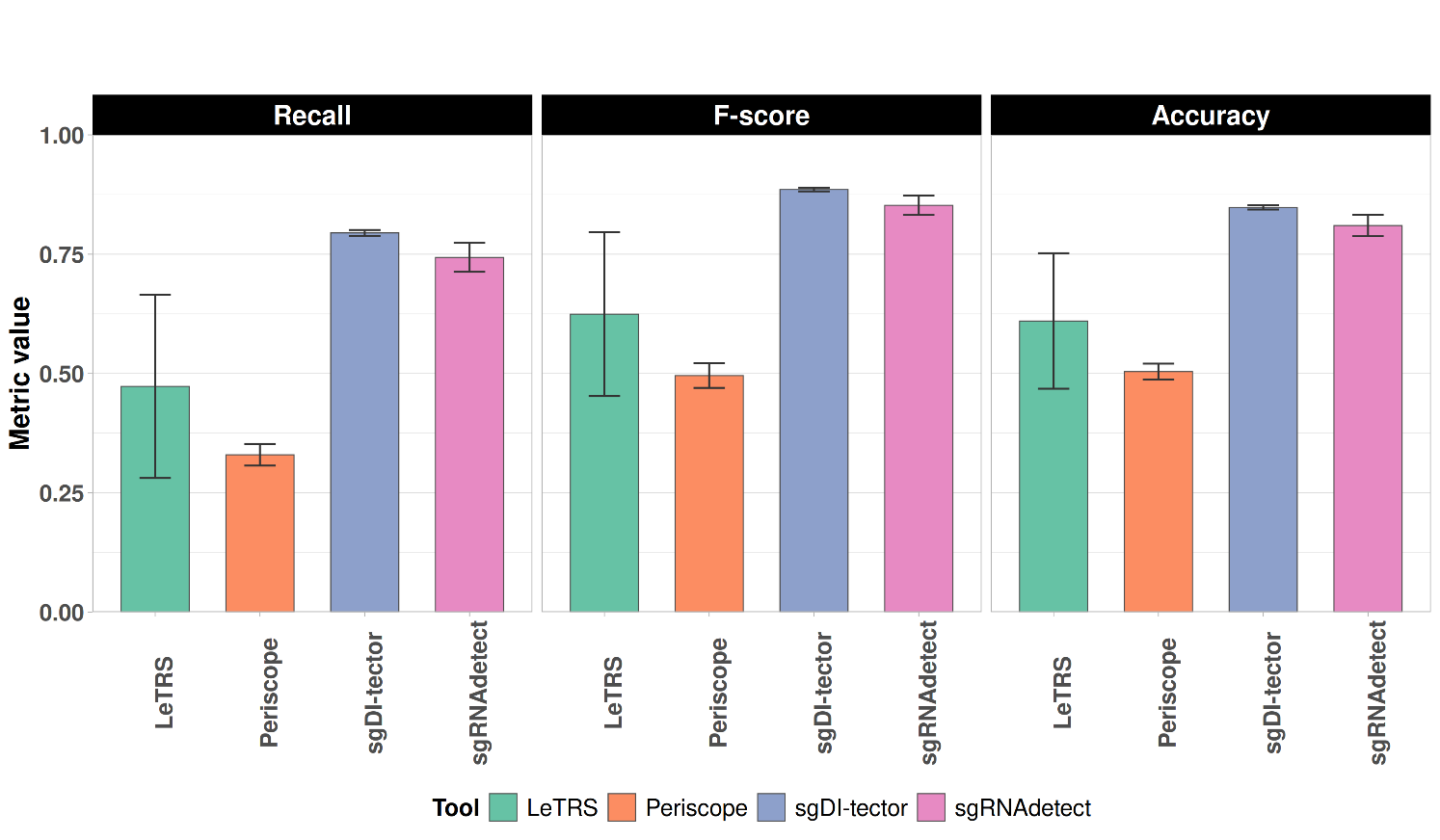
**

**Supplementary Figure 5: Summary of performance metrics for shotgun sequencing datasets.** Bar plots showing the mean ± standard deviation (SD) of Recall, F-score, and Accuracy for each tool across the five mutation conditions (WT, TRS-B mutation, and 1–3 N gene mutations). Precision is not shown as it equals 1 across all tools and conditions (no false positives detected in any dataset). Error bars represent ±1 SD and quantify performance variability attributable to mutation profile. Each bar summarises the five shotgun datasets described in Table 2.


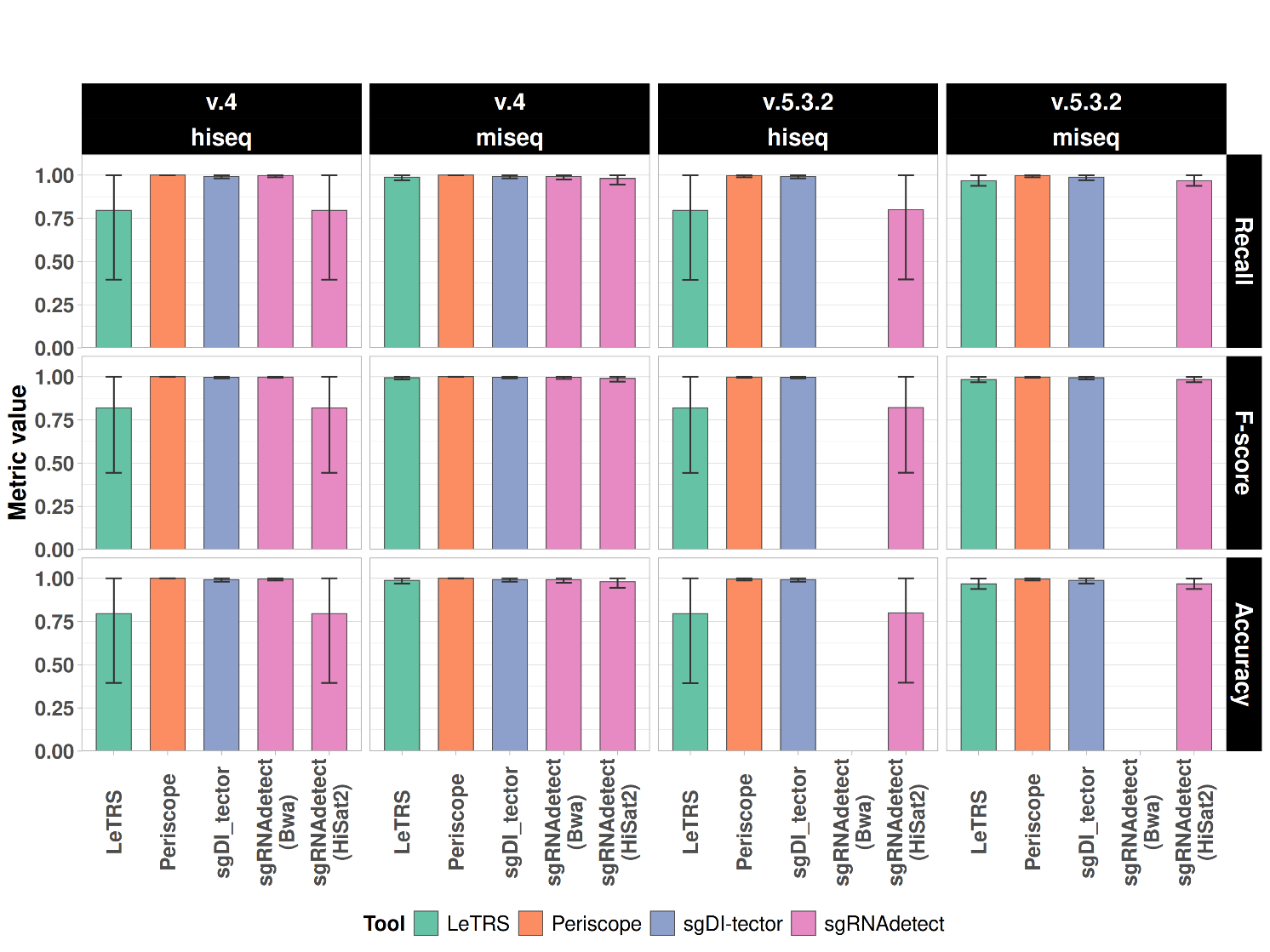


**Supplementary Figure 6: Summary of performance metrics for amplicon sequencing datasets.** Bar plots showing the mean ± SD of Recall, F-score, and Accuracy for each tool and mapper combination, stratified by ARTIC primer scheme version (v.4 vs. v.5.3.2) and sequencing error model (HiSeq vs. MiSeq). Precision is not shown as it equals 1 across all tools and conditions (no false positives detected in any dataset). Error bars represent ±1 SD across the five mutation conditions within each stratum. sgRNAdetect is shown separately for BWA- and HISAT2-aligned data (consistent with Figure 4 of the main manuscript). In this analysis on *in silico* sequence data, sgRNAdetect performance seems to drop when using BWA with ARTIC v.5.3.2 primers. For this reason, we considered HISAT2 as preferential alignment tool in this specific setting (*i.e*., when using sgRNAdetect with ARTIC v.5.3.2 primer scheme on *in silico* sequence data).


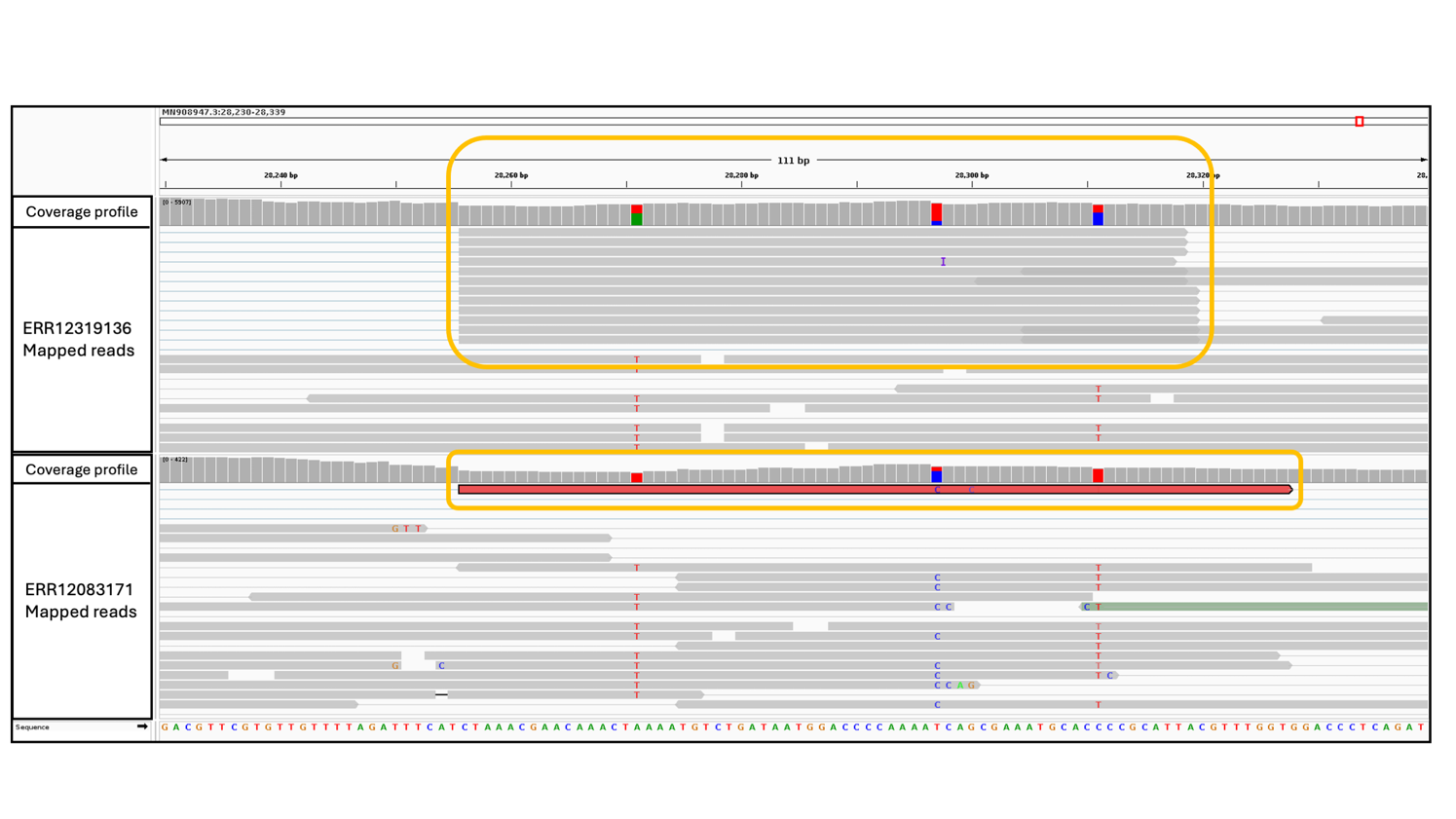


**Supplementary Figure 7: LeTRS-detected sgRNA-supporting reads at the SARS-CoV-2 N gene locus in wastewater samples.** IGV screenshots showing mapped reads at the N gene locus for two samples. The top panel displays sample ERR12319136, in which LeTRS flagged 12 reads as sgRNA-supporting. The bottom panel displays sample ERR12083171, in which a single sgRNA-supporting read was detected. In both cases, the flagged reads (orange boxes) lack the mutations prevalent in the majority of reads in the same sample. This observation may reflect the co-circulation of minority SARS-CoV-2 lineages lacking the predominant mutations, sequencing contamination from previous runs or, consistent with our *in silico* findings, a failure of HISAT2 to accurately map reads carrying multiple mutations, leading these reads to be misaligned and spuriously detected as sgRNA-supporting events.
